# A review of the impact of financing mechanisms on maternal health care in Australia

**DOI:** 10.1186/s12889-019-7850-6

**Published:** 2019-11-21

**Authors:** Haylee Fox, Stephanie M. Topp, Emily Callander, Daniel Lindsay

**Affiliations:** 1 College of Public Health, Medical and Veterinary Sciences, James Cook University, Townsville, QLD 4814 Australia; 2 The Nossal Institute for Global Health, Melbourne School of Population and Global Health, the University of Melbourne, Melbourne, VIC 3010 Australia; 3 School of Medicine, Griffith University, Southport, QLD 4215 Australia

**Keywords:** Health systems, Financing, Maternal health, Medicare, Privatisation, Australia

## Abstract

**Background:**

The World Health Organization states there are three interrelated domains that are fundamental to achieving and maintaining universal access to care - raising sufficient funds for health care, reducing financial barriers to access by pooling funds in a way that prevents out-of-pocket costs, and allocating funds in a way that promotes quality, efficiency and equity. In Australia, a comprehensive account of the mechanisms for financing the health system have not been synthesised elsewhere. Therefore, to understand how the maternal health system is financed, this review aims to examine the mechanisms for funding, pooling and purchasing maternal health care and the influence these financing mechanisms have on the delivery of maternal health services in Australia.

**Methods:**

We conducted a scoping review and interpretative synthesis of the financing mechanisms and their impact on Australia’s maternal health system. Due to the nature of the study question, the review had a major focus on grey literature. The search was undertaken in three stages including; searching (1) Google search engine (2) targeted websites and (3) academic databases. Executive summaries and table of contents were screened for grey literature documents and Titles and Abstracts were screened for journal articles. Screening of publications’ full-text followed. Data relating to either funding, pooling, or purchasing of maternal health care were extracted for synthesis.

**Results:**

A total of 69 manuscripts were included in the synthesis, with 52 of those from the Google search engine and targeted website (grey literature) search. A total of 17 articles we included in the synthesis from the database search.

**Conclusion:**

Our study provides a critical review of the mechanisms by which revenues are raised, funds are pooled and their impact on the way health care services are purchased for mothers and babies in Australia. Australia’s maternal health system is financed via both public and private sources, which consequentially creates a two-tiered system. Mothers who can afford private health insurance – typically wealthier, urban and non-First Nations women - therefore receive additional benefits of private care, which further exacerbates inequity between these groups of mothers and babies. The increasing out of pocket costs associated with obstetric care may create a financial burden for women to access necessary care or it may cause them to skip care altogether if the costs are too great.

## Background

The architecture of health care financing affects how a health system performs and a country’s ability to achieve the goals of universal health coverage for all mothers and babies [1–3]. There are many mechanisms (e.g., tax revenues, non-tax revenues, external grants or loans, out of pocket payments and voluntary health insurance) for financing of maternal health services [1]. However, predominantly relying on public versus private funding sources is considered to be a more progressive method for financing a health system [4]. The World Health Organization (WHO) has stated that countries primarily relying on public sources make greater progress towards universal health coverage [5], although notable exceptions exist. The French health system, for example, with publically subsidised supplementary private health insurance for over 90% of the population has some of the lowest out of pocket costs in the Organization for Economic Co-operation and Development (OECD) and falling [6]. Public revenues enable risk-sharing between the rich and the poor and between those who are healthy and those who are sick in society. Consequentially, this enables health systems to improve access to maternal health services, with financial protection for all. When health systems rely upon private funding sources, and mothers have to pay for health services out-of-pocket, some mothers and babies will not be able to access the health services that they need [5].

Globally, Australia has one of the highest rates of per-capita out-of-pocket healthcare expenditure [7], despite having a universal health insurance scheme (Medicare) in place for over 30 years [8]. Out-of-pocket costs that can be incurred when people access general practitioners, specialists, allied health care services, medical care at private hospitals and pharmaceuticals, causing people to either delay or forego accessing necessary health care, with the greatest financial strain felt by those with lower incomes [9, 10]. This may be particularly felt by those accessing maternal health care, as the out of pocket charges for obstetric related services have increased far more rapidly than other areas of care [11]. The Australian Institute of Health and Welfare (AIHW) stated that in some areas of healthcare there has been a decrease in government financial contributions, resulting in costs being transferred onto individuals in the form of out-of-pocket payments [12].

The WHO states there are three inter-related domains that are fundamental for moving towards universal health coverage, including; raising sufficient funds for health care, reducing financial barriers to access by pooling funds in a way that prevents out-of-pocket costs, and allocating funds in a way that promotes quality, efficiency and equity [13]. Advancements in these three areas will be important factors in determining whether health services are available for everyone, irrespective of ability to pay [13]. Understanding how Australia’s maternal health system is financed is essential for identifying if there are areas of inadequacy within healthcare financing policy that might affect the ability of mothers and their babies to access necessary care. Based on the WHO’s fundamental domains for achieving universal health coverage, this review will explore the funding,^1^ pooling,^2^ and purchase^3^ of maternal health services in Australia.

## Methods

A scoping review and interpretative synthesis drawing on electronic and non-electronic materials was conducted to characterise the current health financing mechanisms of maternal health care in Australia. In this study, we grouped the financing mechanisms under separate headings of ‘Funding’, ‘Pooling’, and ‘Purchasing’, and drawing on both primary and secondary sources asked:

1. What are the mechanisms for funding, pooling and purchasing maternal health care in Australia?
2. How do financing mechanisms influence the delivery of maternal health services in Australia?

Due to the nature of the study question, this study focused on searching primary sources sometimes referred to as ‘grey literature’ as well as peer-review publications. Grey literature includes ‘that which is produced on all levels of government, academics, business and industry in print and electronic formats, but which is not controlled by commercial publishers’ [14]. Some methods for grey literature searches have been described in the literature [15–19], however, no ‘gold standard’ for grey literature have been developed. The Cochrane Handbook, which is an official guide for undertaking systematic reviews, provides insufficient guidance for searching grey literature [20]. In order to ensure transparency of study findings, the authors drew on one methodological study [19], which provided the most comprehensive details for applying systematic review search methods to the grey literature that adheres to the Preferred Reporting Items for Systematic Reviews and Met-Analyses (PRISMA) guidelines [21]. A review protocol was not developed and this review was not registered.

### Eligibility criteria

Documents considered for inclusion in the study were those that were published in English, if they were the most recent version of the document, and contained any information on the funding, pooling, or purchasing of health care in Australia that is applicable to maternal health. The first literature search was conducted between October and December, 2017 and included the time period of 2000 to 2017. The review was updated in July 2019 to include the information from 2018 to the date of the literature search.

### Information sources and searching strategies

The document and source search incorporated three different search strategies. The first two strategies were of the grey literature, which included searching Google search engine (Chrome) and targeted websites. The third search strategy was a traditional systematic review of academic databases.

Due to the nature of the internet, it is impractical to screen all results produced by Google. Google uses algorithms to rank the importance of website pages relevant to the search terms [22], allowing for narrow and specific searching, which was relied upon for producing relevant results. Therefore, the researchers screened the first 10 pages (a total of 100 pages per search). Advanced search engine searching methods that only included specific websites ending in specific suffixes was conducted using the following suffixes:

- :gov.au.
- :edu.au.
- :int.
- :org.

Using these suffixes the following keywords and phrases included in the search were*: Healthcare, costs, fees, Charges, Expenditure, Out of pocket, Healthcare financing, Health policy, Health expenditures, Funding, Healthcare reform, Universal Health Coverage, Resource allocation, Financial management, Federal Government, State and Territory Government, Economics, Maternal Health Services, Pregnancy, Labour, Birth, Obstetric, Midwife, Model of Care, Hospital, Delivery of Health Care, Revenue raising, Tax, Pooling, Funding, Purchasing, Medicare and Australia*. The keywords were combined in different formats using OR and AND. An example of a search strategy used in the Google search was:

Medicare AND Australia:gov.au.

The second search strategy involved the first author searching specific websites of applicable health, research, and government organisations. Firstly, the author searched Google to establish websites that contained relevant information for addressing the research question. Each of the websites identified was then hand searched via the websites search bar. The grey literature search was conducted between October 23rd and December 20th, 2017.

The third search strategy was of academic databases. The first author searched titles, abstracts, and keywords in CINAHL, Informit, Cochrane Library, and Scopus databases during the month of November 2017 to obtain peer-review journal articles that met the inclusion criteria. The same keywords used in the first search strategy were used in the database search by combining different words using “OR”, “AND” and Truncation (*). A search strategy used in Scopus is presented in Table 1. Manuscripts were excluded at this stage of the search if they were unrelated to the Australian healthcare system or if a more relevant manuscript was available detailing similar information. After title and abstract screening, the full texts were imported into Endnote and duplicates were removed.

**Table 1:** Search strategy, Scopus

	Search strategy	Results
1	TITLE-ABS-KEY (“Federal Government”)	30,049 document results
2	TITLE-ABS-KEY (“Health expenditure*”)	20,459 document results
3	TITLE-ABS-KEY (austral*)	565,915 document results
4	(TITLE-ABS-KEY (“Federal Government”)) AND (TITLE-ABS-KEY (“Health expenditure*”)) AND (TITLE-ABS-KEY (austral*))	7 document results

### Eligibility assessment and study selection

**Fig. 1:**
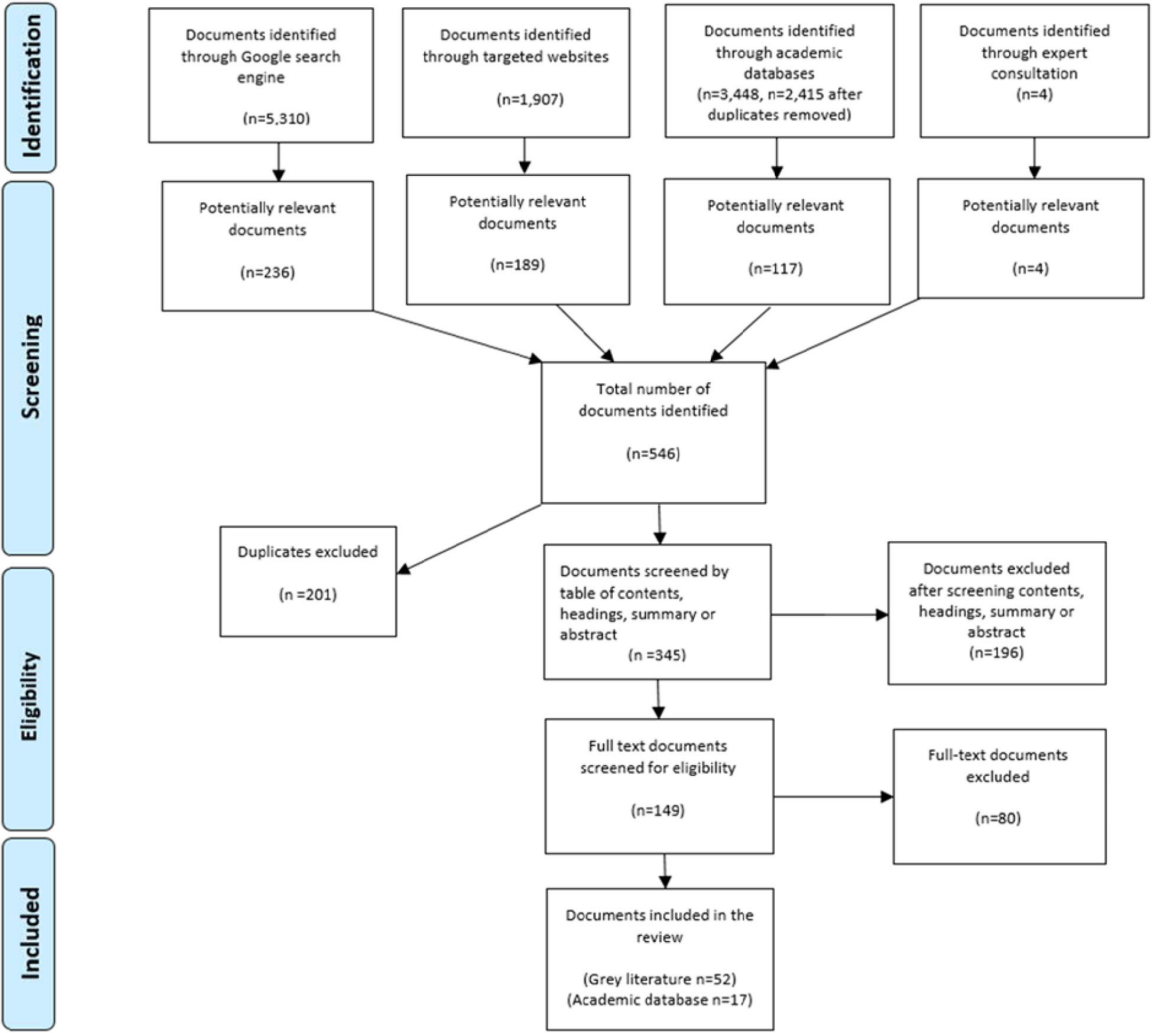
PRISMA Flow Diagram

The PRISMA flow diagram was also applied to the grey literature search (Fig. 1). It is uncommon for grey literature to have abstracts [15], therefore, executive summaries, table of contents or subheadings were screened. The first author approached this stage in a conservative manner and continued screening the document or web page further to assess for relevance if the review question was not explicitly addressed, but still warranted further investigation. The details of the documents and web pages were manually entered into an Excel file. The information included in the data extraction was the source organization, title, date published, URL and any information relating to the funding, pooling or purchasing of maternity care services in Australia were entered under these headings. The final documents were downloaded in full to ensure they addressed the research questions. A total of 52 documents and web pages in the grey literature search were included in the review. The combination of the three search strategies resulted in a total of 69 documents and web pages. The researchers found that if they had of relied solely on academic databases for the source of information 75% of the manuscripts would not have been identified. Refer to Additional file 1 for all documents included in this review.

### Data collection process and synthesis of results

Following basic demographic information about manuscript, date, title, author and sources, data extraction was structured around the two research questions, and included: characteristics of general health system funding, pooling and purchasing; maternal health service models; maternal health-specific examples of funding, pooling and purchasing in Australia; current services costs; and identifiable trends. Categories were used to produce descriptive and subsequently analytical summaries that were refined through several phases of discussion and writing among all authors. Documents that did not address (either explicitly or inexplicitly) the funding, pooling or purchasing of maternal health services in Australia were excluded at this stage. Those that did, were then extracted into the data extraction tool.

## Results

We present the results in three sections. First, given the absence of such in either peer-review or grey literature to-date, we briefly summarise the funding, pooling and purchasing mechanisms in the Australian health system at-large. Second, we describe the models of maternity care in Australia and their relationship to the funding, pooling and purchasing mechanisms. Third, we reflect on three emerging trends in maternal health care that appear to be linked to financing mechanisms, as synthesised from the literature.

### Section 1: financing mechanisms in the Australian health system funding

#### Government funding

**Fig. 2:**
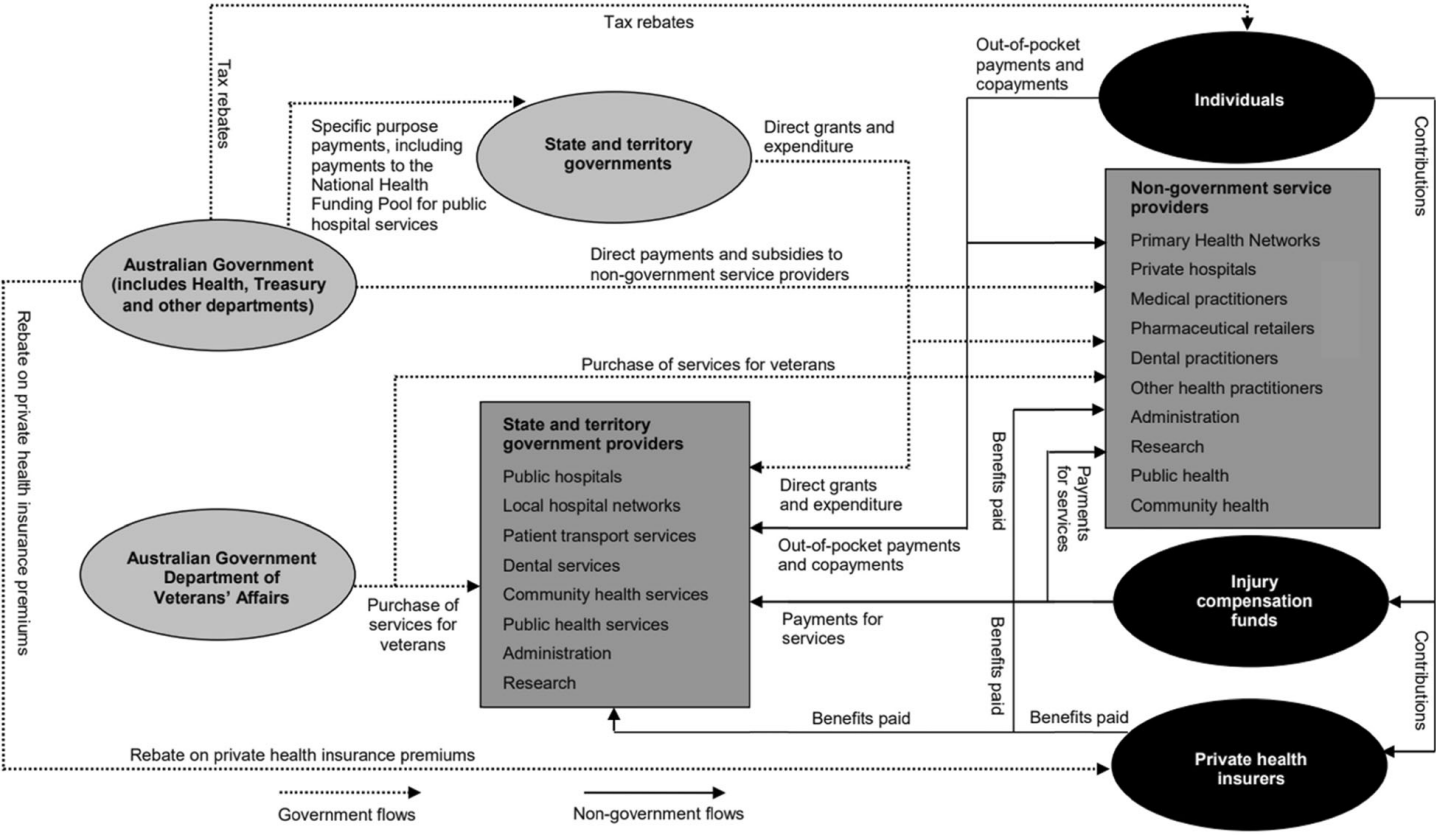
Source: The Australian Institute of Health and Welfare [1]

Healthcare funding mechanisms in Australia are complex and determined by government and non-government sectors. Government sectors include the Federal,^4^ state and territory governments, and in some jurisdictions, local governments. The non-government sectors primarily include individuals, private health insurers, third-party motor vehicle insurers, workers compensation and funding for research from non-Government organisations [24]. Figure 2 provides an illustration of the funding sources and relationships and the types of products that are financed.

The *National Health Reform Agreement*, which sets out Australia’s health care funding rules, was established in 2011 between states, territories and the Federal government to guide an effective partnership for funding, pooling and purchasing health care goods and services. The aim of the agreement was to establish shared incentives for all levels of government to make better use of resources [23, 25]. The agreement recognises that the states and territories are the system managers of public hospitals and the Federal Government has full funding and program responsibility for General Practitioner (GP) services, and primary health care. The health financing arrangements of the agreement include block funding and Activity Based Funding (ABF). Block funding is a Federal Government funding system for public hospitals whereby a fixed amount is provided to public hospitals based on population size and the previous year of funding. ABF is a way of funding hospitals where the hospitals get paid based solely on the number, mix, and casemix of patients they treat. If a hospital treats more patients, they receive more funding [26]. However, under the current ABF arrangements, the Federal Government will not increase payments to each state and territory by more than 6.5%, limiting the potential for hospitals to increase revenue by increasing case-load by that amount [27].

All levels of government source funds to finance the health care system from various types of taxes and levies [24]. The Federal Government is the major tax collector (raising 81% of Australia’s total tax funds), and it divides the funds among the lower levels of government with 50% going to the state and territory and local governments. Funding is also received by non-tax revenues such as minerals, gas, and petroleum, which can be allocated to health [28]. The funds from taxation and non-tax revenue are then used by the Federal Government to pay for block funding and ABF towards the states and territories by depositing the funds into the National Health Funding Pool [29].

Levies are an additional charge that can be collected by any level of government to fund health care [30]. The Federal Government has imposed several levies to collect funds to finance Medicare, including the ‘Medicare Levy’ and the ‘Medicare Levy Surcharge’ [31]. The Medicare Levy is currently set at 2% of taxable income on individuals that earn above A$21,655 per annum [32], making it a ‘flat tax’ as both high-and-low income earners are contributing the same portion of their income [33]. The Medicare Levy Surcharge imposes a further 1–1.5% on high-income earners who do not have private health insurance and earn above A$90,000 per annum for singles and A$180,000 per annum for families [31]. The purpose of the Medicare Levy Surcharge was to encourage people to uptake private health insurance and reduce the burden on the public health system [31].

#### Non-government funding

Out-of-pocket costs incurred by individuals made up A$24.4 billion out of the total A$140.2 billion spent on health care in 2011–12, which is more than double the A$11 billion spent in the previous decade. The proportion of total health expenditure funded by individual out-of-pocket costs during this time remained relatively unchanged (17.5% in 2002 and 17.3% in 2012) [34]. Private health insurance¸ which is held by 57.1% of Australians aged 18 years and over [35], produces two costs; insurance premiums and out-of-pocket costs to cover medical treatment that is not covered by either Medicare or the private health insurer. ‘Gap payments’, which are payments made by the individual for either hospital or medical charges that are greater than what the private health insurer covers, vary between different private health insurers, with the average gap payment for in-hospital treatment being A$316 (March 2019) [36].

### Pooling

Funding from both Government and non-Government sources are pooled separately. Expenditure by the Federal Government Department of Health, Medicare and the Pharmaceutical Benefits Scheme come from general revenue. Levies are paid into general revenue and are not hypothecated to health. Non-Government resources of health expenditure are paid to health providers either through Private Health Insurance or out-of-pocket payments.

#### Inter-governmental pooling

Funding that is pooled into the National Health Funding Pool is managed by an Administrator who is distinct from any level of government. They are responsible for ensuring that funds are deposited and administered as per the National Health Reform Agreement and for overseeing payments into and out of the pool account for each state and territory [37]. Maximising the system’s capacity to redistribute resources is central to achieving the goals of financial protection and equity in service use; in this way, service use can be driven by health needs, rather than an ability to pay.

#### Government-private-sector pooling

Pooling in the private sector is achieved via Government-subsidized premiums. The ‘Australian Government Private Health Insurance Rebate’ is an income-tested rebate that the Australian Government provides people to help cover the cost of their private health insurance premiums. The percentage that is rebated is anywhere from 0% for those who earn greater than A$140,001 per single or $280,001 per family, up to 38% for those on lower incomes [38]. The Lifetime Health Cover Private Health Insurance was introduced with the objective of increasing the uptake of private hospital insurance earlier in life. The Lifetime Health Cover enforces penalties in the form of premium loadings if the health insurance is not purchased by the age of 31 [39]. This policy, introduced in 2001 has been shown to be a key driver of the current increase in the percentage of people with private health insurance in Australia [40].

Voluntary health insurance should spread risk and make access to health care more affordable. However, insurance premiums, even where subsidised, remain a key barrier to uptake of such insurance for those in lower-income brackets. In Australia, for example, those with private health insurance are made up of wealthier [41], urban [42], non-Aboriginal or Torres Strait Islander [43] people. Therefore, pooling money into voluntary health insurance schemes such as private health may not maximise the redistributive capacity of public revenues.

### Purchasing

#### Private hospitals

Australia has a total of 1359 public and private hospitals (747 and 612 respectively) [12]. Private hospitals are owned and operated by the private sector, however, they are licensed and regulated by governments. Hospitals in the private sector consist of not-for-profits and for-profits, with different incentives and therefore, different market behaviours. The Private Health Insurance industry is highly concentrated with only 5 funds accounting for more than 80% of all policies, with almost 70% of the industry now operating on a for-profit basis [12].

#### Public hospitals

Purchasing public health services in Australia involves both levels of government, creating a complex set of overlapping and fragmented responsibilities [44]. Each state and territory has its own government and holds responsibility for public hospital care and community health services within its jurisdiction. Money is received by the states and territories via the National Health Funding Pool and then each state and territory decides how to spend their money on purchasing health services. The states and territories operate public hospitals, however, funding them is a joint responsibility of both Federal and state governments. The Federal government is solely responsible for purchasing benefits through Medicare for health services such as out-of-hospital medical care and in-hospital private medical care, and for the Pharmaceutical Benefits Scheme (see below) [44]. Medicare itself does not deliver healthcare but rather it purchases healthcare services for those covered by the scheme, which allows for free treatment for public patients in public hospitals and subsidises private patients in public hospitals (75% of the schedule fee). Federal and state and territory governments are also responsible for funding and delivering health and medical research, Aboriginal and Torres Strait Islander specific health services, public health initiatives, and community health services. Local governments provide community-based health services alongside contributing to public health and health promotion initiatives, such as child and maternal health services [45].

#### Pharmaceuticals Benefits Scheme

Medicines are subsidised by the Federal government under the Pharmaceuticals Benefits Scheme (PBS) [12]. The PBS schedule lists all of the medicines that can be administered to all Australian residents that hold a Medicare card at a government-subsidised price [46]. Under the PBS, the cost incurred by the patient varies, depending on the difference between the schedule fee and the actual cost of the medication with a maximum payment of A$38.30 for general patients and A$6.20 for people with a concession card [47]. Safety net thresholds exist to reduce the financial burden for those that require a substantial amount of medications. The safety net threshold is A$378.00 per annum for concession card holders and A$1494.90 for all other patients. After reaching the safety net threshold, general patients pay for any further PBS prescriptions at the concessional payment rate and concession card holders face no further charges for medications for the remainder of the calendar year [46]. If a medication is not listed on the PBS schedule, the patient has to pay the full price for the prescription [48]. Pharmaceuticals for public patients in public hospitals are typically provided for free [12]. However, Australians pay almost four times more than the best international prices for a range of out of hospital prescription medicines, with 6% of patients delaying or forgoing necessary medication due to cost [49].

#### Primary health care

General Practitioners (GPs) are considered the primary point of medical care and the gatekeeper to the rest of the health system as all specialist care requires a GP referral. Medicare purchases out-of-hospital medical services and therefore provides some benefits under the Medicare Benefits Schedule (MBS) for services such as consultations with medical specialists and general practitioners (80% or 100% of the schedule fee). Under the MBS, patients will receive a ‘rebate’, which is based upon a proportion of the schedule fee covering each type of service. For example, when a woman receives a pathology test to confirm pregnancy it has a schedule fee of A$10.15 and the benefit of the fee is 75% or A$7.65 [8]. There are three potential fee options for a GP consultation: the doctor bulk bills the patient and Medicare rebates 100% of the Schedule fee leaving the patient with no out-of-pocket costs; the doctor bulk bills the patient but the fees charged for the service are greater than the Medicare Schedule fee leaving the patient with a ‘gap fee’; or the doctor does not bulk bill and the patient is left to cover the entire consultation fee [50]. The decision to bulk bill a patient is at the discretion of the doctor. If a doctor decides to bulk bill it means their payment for the service provided will be either 85 or 100% (depending on the type of service provided) of the Medicare Schedule fee. Greater than 80% of all GP consultations are paid for via bulk billing arrangements under Medicare. However, many doctors charge above the schedule fee leaving patients with a ‘gap fee’, whereby the doctor receives a payment from both Medicare and the patient [51]. GPs primarily work in private practices, where they receive a fee for service payment [50].

As part of Medicare, the ‘Original Medicare Safety Net’ (OMSN) was introduced with the aim to provide a 100% financial rebate to individuals accessing out-of-hospital services once an annual threshold is met. The Extended Medicare Safety Net (EMSN), which works in conjunction with the OMSN, also forms part of Medicare with the aim to provide a higher Medicare benefit for out-of-hospital health care costs for people with ongoing health needs. Once the annual threshold of out-of-pocket costs has been met, Medicare will pay for 80% of any future out-of-pocket costs for out-of-hospital Medicare services for the remainder of the calendar year. Due to unregulated provider fees in Australia [40], private providers can charge well above the schedule fee. Even with the 80% EMSN fee coverage, high fees charged by private providers mean that individuals may be left with a significant difference to cover as an out-of-pocket cost [52].

### Section 2: models of maternity care in Australia

As with health care generally, the arrangements underpinning maternity services in Australia are complex and achieved through a mix of Federal, state and territory and private funding and delivery via state and territory government providers’ and Non-government service providers (Fig. 2). The Federal Government funds maternal services through the MBS and PBS, state governments through the National Healthcare Agreement, private health insurance via the private health insurance rebate and through other specifically targeted programs including Indigenous maternal and child health programs [53]. Limited information is available on the costs of providing maternity care in Australia, which restricts the ability to revise maternity service funding [54]. The AIHW reported that the total expenditure on maternity care in 2004–05 was $1672 million. Of this, $1538 million was spent on hospital-admitted services associated with births taking place in a hospital and $134 million was spent on neonatal care [55]. State and territory and local governments fund and deliver a range of *community* health services such as antenatal and postnatal parenting support, breastfeeding programs, immunisation services, and health promotion programs targeted at women during the perinatal period. However, a comprehensive national picture of community health services is not available due to a lack of statistical information being collected [12].

Maternal health care in Australia includes antenatal, intrapartum, and postnatal care for mothers and babies up to 6 weeks after birth [56]. A review of Australia’s maternity services (2010) [54] found that women were dissatisfied with the current system and the choices that were or were not available to them. Many women who took part in the review indicated a preference to receive care from midwives. In Australia, a range of different models of maternity care are available [57–60] (Table 2). The availability of maternity care models within the public and private system and the characteristics of the model can differ between states, between levels of rurality and between individual health services.

**Table 2:** The major Model Categories from the Maternity Care Classification System [1]. Source: University of New South Wales and Australian Institute of Health and Welfare

Model of care	Characteristics
Private obstetrician (specialist) care	Antenatal care provided by a private specialist obstetrician. Intrapartum care is provided in either a private or public hospital by the private specialist obstetrician and hospital midwives in collaboration. Postnatal care is usually provided in the hospital by the private specialist obstetrician and hospital midwives and may continue in the home, hotel or hostel.
Private midwifery care	Antenatal, intrapartum and postnatal care is provided by a private midwife or group of midwives in collaboration with doctors in the event of identified risk factors. Antenatal, intrapartum and postnatal care could be provided in a range of locations including the home.
General Practitioner obstetrician care	Antenatal care provided by a GP obstetrician. Intrapartum care is provided in either a private or public hospital by the GP obstetrician and hospital midwives in collaboration. Postnatal care is usually provided in the hospital by the GP obstetrician and hospital midwives and may continue in the home or community.
Public hospital maternity care	Antenatal care is provided in hospital outpatient clinics (either onsite or outreach) by midwives and/or doctors. Care could also be provided by a multidisciplinary team. Intrapartum and postnatal care is provided in the hospital by midwives and doctors in collaboration. Postnatal care may continue in the home or community by hospital midwives.
Public hospital high-risk maternity	Antenatal care is provided to women with medical high risk/complex pregnancies by maternity care providers (specialist obstetricians and/or maternal-fetal medicine subspecialists in collaboration with midwives) with an interest in high-risk maternity care in a public hospital. Intrapartum and postnatal care is provided by hospital doctors and midwives. Postnatal care may continue in the home or community by hospital midwives.
Shared care	Antenatal care is provided by a community maternity service provider (doctor and/or midwife) in collaboration with public hospital doctors and midwives under an established agreement and can occur both in the community and in hospital outpatient clinics. Intrapartum and early postnatal care usually takes place in a public hospital by hospital midwives and doctors often in conjunction with the community doctor or midwife (particularly in rural settings).
Combined care	Antenatal care is provided by a community maternity service provider (doctor and/or midwife) in the community. Intrapartum and early postnatal care is provided in the public hospital by hospital midwives and doctors.
Team midwifery care	Antenatal, intrapartum and postnatal care is provided by a small team of rostered midwives (no more than eight) in collaboration with doctors in the event of identified risk factors. Intrapartum care is usually provided in a public hospital or birth centre. Postnatal care may continue in the home or community by the team midwives.
Midwifery Group Practice caseload care	Antenatal, intrapartum and postnatal care is provided within a publicly-funded caseload model by a known primary midwife with secondary backup midwife/midwives providing cover and assistance with collaboration with doctors in the event of identified risk factors. Antenatal care and postnatal care is usually provided in a public hospital, community or home with intrapartum care in a hospital, birth centre or home.
Remote area maternity care	Antenatal and postnatal care is provided in remote communities by a remote area midwife (or a remote area nurse) or group of midwives sometimes in collaboration with a remote area nurse and/or doctor. Antenatal care may also be provided via telehealth or fly-in-fly-out clinicians in an outreach setting. Intrapartum and early postnatal care is provided in a regional or metropolitan hospital (involving temporary relocation prior to labour) by hospital midwives and doctors.
Private obstetrician and privately practising midwife joint care	Antenatal, intrapartum and postnatal care is provided by a privately practising obstetrician and midwife from the same collaborative private practice. Intrapartum care is usually provided in either a private or public hospital by the privately practising midwife and/or private specialist obstetrician in collaboration with hospital midwifery staff. Postnatal care is usually provided in the hospital and may continue on in the home, hotel or hostel by the privately practising midwife.

‘Doctors’ include specialist obstetricians, GP obstetricians and obstetricians in training

Ninety-three per cent of mothers receive care through one of four models; private obstetric care (31.8%); combined maternity care (24.3%); public hospital maternity care (22.4%), and shared maternity care (14.2%) [54]. Less commonly accessed models include private midwifery care, and team and caseload midwifery care.

Pregnant women who are screened as having a ‘low risk’ pregnancy and want to receive care as a public patient usually receive advice from their GP to book in at their closest hospital that has maternity services available. In public hospital care, it is unlikely that mothers will receive the same doctor or midwife at each antenatal check-up. Additionally, the doctors and midwives that attend the antenatal appointments are not likely to be the practitioners that attend the birth [61]. As a public patient, mothers do not have a choice of practitioner but fees and expenses are typically low or provided for free if the mother holds a Medicare card.

If a woman is considered a ‘low risk’ pregnancy and she can access a public health service that provides either shared or combined care, she can elect to receive one of these models of care [57]. In shared and combined care, the public hospital receives funding for each inpatient hospital event through either ABF or block funding and the doctor receives funding for each occasion of service delivered through the MBS and from the mother for any gap payments. The doctor can also charge a once-off management fee under the MBS, with the woman again liable for any gap payments. Mothers who access the shared or combined models of care may incur some out-of-pocket fees as doctors and midwives may impose these costs and the amount charged can vary. Although Medicare provides rebates to mothers to cover a portion of the cost of care when they access non-public services, medical provider fees are unregulated [40], leaving patients to pay the “out-of-pocket” cost difference between the providers’ fee and the Medicare rebate [62]. The majority of out-of-pocket costs that mothers incur for maternity services are related to specialist medical services (e.g. obstetric services) [54], as only a small portion of such services are bulk billed^5^ [34].

If a woman can afford to, she can also choose to receive wholly private obstetric care. The private obstetric model of care allows for choice of obstetrician and typically has shorter appointment waiting times. However, due to the cost of private health insurance and not all medical items being covered by the insurer (such as out of hospital costs) doing so can be quite expensive. In this model of care, the private obstetrician will receive fees for his/her service via the MBS and from the woman for any gap payments [63]. Most OECD countries have abandoned this way of spending public funds due to the rising costs associated with private health and the inequities caused by having a ‘two-tiered’ health system [64]. Australians continue to experience the repercussion of Private Health Insurance reforms, with only those who can afford private health insurance receiving more timely access to health care services [34, 41].

Continuity of care, whereby a woman receives perinatal care by the same midwife or team of midwives, is considered ‘gold standard’ for mothers during pregnancy and childbirth as it is known to improve birth outcomes for both the mother and baby [65, 66]. In Australia, Midwifery Group Practice Caseload Care is the model of care that is most aligned with continuity of care. The level of continuity varies both between and within different models of care due to variations in the characteristics of models of care between individual health services [67]. Women may access continuity of midwifery care in Australia by either being allocated to official models of maternity care whereby women receive continuous care from a midwife or a team of midwives – ‘Midwifery Group Practice Caseload Care’ and ‘Team Midwifery Care’ in a public hospital or by engaging a private midwife to provide care, and still giving birth in a public hospital. The terms “continuity model” or “continuity model of care”, although not the official terms for models of care, they are terms that are commonly used in maternity care, particularly in the midwifery field [68]. In a public midwife continuity model, the public hospital receives funding for each hospital inpatient event through either ABF or block funding; with the private midwife model, the public hospital still receives funding for each inpatient event, but the private midwife will also receive funding through the MBS, and from the woman for any gap payments.

### Section 3: trends in financing and maternity care in Australia

In reviewing the financing of the various maternity models outlined above, we identified three trends. First, a trend towards privatisation of maternity care; second increasing medicalisation of birth; and third, a concurrent limiting of Australian mothers’ choice to access midwifery care. All three trends are likely to have contributed to the rising costs of maternal healthcare to both individuals and the health system.

#### Privatisation and rising costs of maternity care

Currently, 26% of mothers who give birth in Australian hospitals do so in a private hospital under the care of a private obstetrician and are thus liable for some type of gap payment [69]. The evidence reviewed in this study suggests that pooling funds through private health care providers has weakened the efficiency of the publically funded health system by facilitating market-driven price-setting among private health care providers and insurance companies. Following the introduction of the EMSN, there was a substantial rise in consultation fees charged by privately practising obstetricians for antenatal attendances [56], with out of pocket charges for obstetric services delivered outside hospitals rising 1035% between 1992 and 2016, even after adjusting for inflation [11]. The costs to individuals of this trend are substantial as fees incurred out of hospital are not covered by private health insurance. Even for in-hospital out of pocket fees, where private health insurance may pay for some or all of the gap [36], women may be left vulnerable to large out of pocket fees if their private health insurance does not cover the full amount [70]. For example, although the Government schedule fee for an obstetrician consultation in Australia is A$85.55 [52], the *average* (unregulated) fee being charged for an in-hospital obstetrician consultation in Australia in 2017 was A$781.07 [11]. Since the benefit that mothers may claim for this service is calculated as 75% of the government *scheduled* fee (i.e. $64.20) the average gap payment (which is the previously mentioned *total average* $781. 07 fee for the consultation minus the $64.20, which is 75% of the government *scheduled* fee) for mothers attending a single private obstetrician consultation in Australia is $716.87 [71]. A frequently articulated concern regarding private health insurance is the lack of disclosure about the total out-of-pocket costs that will be incurred, with individuals being left with high and unexpected out-of-pocket costs [72]. In response to such complaints, a key private health insurer is trialling a no-gap fee pregnancy program [73]. However, a lack of transparency, inadequate informed financial consent, and uncertainty around whose responsibility financial consent is (between the physician or private health insurer), are recurring complaints by individuals left with high out-of-pocket costs on top of their private health insurance premiums [40, 74].

The costs to the health system are similarly large, with care for reproductive and maternal health costing $7,711,415, 988 (2015–2016) [75]. Between 2003 and 2008, the amount of Federal Government MBS funding for obstetric services climbed 174% from $77 million to $211 million. During the same time period, the number of babies born only increased by 17% from 256,925 to 296,925 [76, 77]. The increased charges associated with providing obstetric care has been absorbed by public funds with a considerable portion of total MBS funding for obstetric services channelled through the EMSN [54]. Of that $134 million increase, approximately $130 million was due to MBS item number 16590, for the ‘Planning and Management of Pregnancy’, which was claimed for services provided by privately practising obstetricians [54]. EMSN payments for obstetric services made up for 31% of total safety net expenditures on all healthcare in 2008 [54] and were paradoxically shown to be larger in areas with high median family income and lower overall health care needs [78].

While public hospitals are managed by state and territory governments, most out of hospital services are delivered by private providers [12]. Therefore, in a private obstetrician led care model, the private obstetrician will receive funding through the MBS for any services delivered, as well as from the woman for any gap payments. There are a number of MBS items that cover post-partum pregnancy care mainly catering to mothers who need medical complications addressed immediately after birth [79].

#### Medicalisation of childbirth

The introduction of Private Health Insurance Incentives Scheme was associated with a decrease in public birth rates and an increase in private birth rates [80]. Simultaneously, there has been an increase in use of medical tests and procedures (for example, episiotomies, epidural, induction of labour, forceps and vacuum extraction) within perinatal care [81, 82], as obstetric involvement, and the use of medical interventions during pregnancy and childbirth have become routine even in low-risk pregnancies [83]. Australia has also seen a decrease in vaginal deliveries from 51.9% in 2004 to 47.1% in 2013, and an increase in caesarean sections both in the public and private sector [84]. Caesarean sections for women giving birth for the first time in Australia have increased from 31.7 to 38.2% in the private sector and 20.4 to 25.8% in the public sector between 2000 and 2015 [85]. This is despite private sector clients generally coming from ethnic, socio-economic and geographic backgrounds with lower rates of maternity-related risk factors that would indicate the need for medical intervention [80, 86]. Caesarean sections are not only more costly than a vaginal delivery ($9603 per cesarean delivery with minor complications, compared with $4211 for a vaginal delivery with minor complications, 2014–15) [87], but they are associated with an increased likelihood that the mother or baby will experience poorer birth outcomes, and increased likelihood that the mother will require a repeat caesarean section for a subsequent birth [88, 89], producing further costs to both individuals and the healthcare system [83].

Although increased medicalisation of childbirth has seen a significant rise in the cost of obstetric services in Australia [11], the full costs are unknown. Unlike some countries [90–92], Australia currently does not monitor the costs associated with the “burden of disease” resulting from maternal health system performance, such as the short-and-long-term costs associated with high rates of obstetric interventions.

#### Women’s choice to access midwifery care

Women who receive midwifery continuity of care models are less likely to have an instrumental birth and more likely to experience a normal vaginal birth [65, 66, 93]. Furthermore, the evidence suggests that for women who receive this model of care, enhanced patient satisfaction during pregnancy and childbirth, with the feelings of greater preparedness for birth and parenting, alongside reduced health care costs being experienced [65]. In 2009, it was suggested an extension of Australia’s Federal Government funding to midwives as primary maternity care providers who are crucial to improving access to evidence-based maternal health care [54]. Despite this, Medicare funding for midwifery services (first introduced in 2006) is still only provided to eligible privately practising midwives working in collaboration with a specified medical practitioner [94] and in specifically prescribed circumstances such as in remote settings where no obstetrician is available [54]. Australian women may access midwifery care through public hospitals and birthing centres, but the supply does not meet the current levels of demand with public hospitals in many locations not offering, or only offering limited access to this model of care [54]. Where women wish to have guaranteed access to continuity of midwifery care, they must access it privately and cover the associated out-of-pocket costs without rebate. The limited role of midwives has been found to have consequential restrictions on women’s choice of care during the perinatal period [54].

## Discussion

This study fills an important gap in the literature by characterising the current health financing mechanisms in Australia and highlighting some concerns relating to their impact on maternal health care. The main concerns identified include increased privatisation and associated rising costs to the system and to individuals; increased medicalisation of birth; and limited access to gold-standard midwifery continuity of care.

The study results demonstrate the dominant combination of ABF and fee-for-service funding models can create an incentive for delivering ‘volume’ of maternal care, rather than the quality of care since a hospital or individual provider is financially rewarded for every occasion of care [62, 71]. The more occasions of care, the more money is received by service providers or institutions, regardless of the outcomes for the mother. This incentive exists in both the public and private system, although in the private system and for out-of-hospital services the incentive may be larger since fees are unregulated [95], and providers operate on a for-profit basis.

Our results also suggest that ABF and fee for service funding models combined with government advocacy for private health insurance could be indirectly contributing to a trend of increasing medicalisation of childbirth. Government reforms that have advocated for the uptake of private health insurance [39], and concurrent pooling of public funds to subsidise private healthcare [38], have encouraged many Australian women to seek private care through a private provider, which have demonstrated higher rates of obstetric interventions. Medical intervention in childbirth attracts a higher payment from the government via ABF in the public system or, in the private system a high payment from some combination of the insurer and client (fee for service).

Lastly, our results indicate that despite midwifery continuity of care models costing less, and having better outcomes for both mothers and their babies [65, 66], the current financing mechanisms actively restrict access to this option. Models of care that encompass midwifery continuity of care characteristics are available in public hospitals, but demand easily outstrips supply [54]. Continuity of care from a midwife or team of midwives is otherwise only available under a private model of maternity care – including paying a midwife – and incurring substantial out of pocket costs, making it inaccessible to many [54]. The current financing mechanisms contribute to this effective restriction on affordable continuity of midwifery care, by directing a large proportion of public maternal health resources into private funding (through MBS subsidies to private obstetricians) [54] and pooling (through the Private Health Insurance Incentive Scheme) [38, 39] of maternal health care. In health systems in other countries such as New Zealand [96], Canada [97], the Netherlands [90], and Britain [91], health financing policy directs funding towards primary health providers such as midwives in community-based services.

### Limitations

This study was based exclusively on document review. It represents our best attempt to interpret current trends and the influence of financing mechanisms on them. However, questions relating to the exact manner and combination in which financing mechanisms are influencing policy and organisation decisions regarding maternity care in all geographical settings in Australia remain and should be the focus of further study. In addition, this review included documents from think tanks, politicians and position statements with views influenced by individual and institutional agendas.

## Conclusion

In summary, there is currently an unequal distribution of maternal health care resources among population groups with those who are financially, ethnically and geographically marginalised experiencing the greatest disadvantages. A combination of Federal policy reforms and unregulated medical fees allows for increasing privatisation and cost-shifting onto mothers who access the healthcare system, with growing costs taking place at both an individual and system level. Financing mechanisms that incentivise volume as opposed to quality of care can mean health services and care providers are not motivated to deliver woman-centred health outcomes.

Although midwifery continuity of care models are more cost-effective and have been demonstrated to produce better health outcomes for both mother and baby, the current financing arrangements leave mothers with limited choice over the type of care they receive. These financing arrangements are inefficient and could be contributing to the increasing medicalisation of maternity care. Specific research is needed to better understand the influence of financial, institutional and political levers shaping the delivery and uptake of different maternity models in twenty-first century Australia.

## Supplementary information

**Additional file 1.** Documents included in the review.

## Data Availability

All data is contained within the manuscript file.

## Abbreviations

ABF: Activity Based Funding
AIHW: Australian Institute of Health and Welfare
EMSN: Extended Medicare Safety Net
GP: General Practitioner
MBS: Medicare Benefits Schedule
OECD: Organization for Economic Co-operation and Development
OMSN: Original Medicare Safety Net
PBS: Pharmaceuticals Benefits Scheme
PRISMA: Preferred Reporting Items for Systematic Reviews and Met-Analyses
WHO: World Health Organization

## Footnotes

1 Funding refers to government policies that are in place to raise revenues to pay for the operations of the health care system

2 Pooling refers to the accumulation of funds on behalf of the population for transfer to health care providers

3 The purchasing of health services refers to the allocation of pooled funds to health care providers that deliver health care goods and services

4 The Government of the Commonwealth of Australia (also referred to as the Australian Government, the Commonwealth Government, or the Federal Government) operates under the Australian Constitution, which defines how the government can pass laws [23].

5 When the provider bills Medicare directly and accepts the Medicare benefit as full payment for their service and the individual does not have any out-of-pocket costs as a result of that appointment.
