# Supplementary material for: A review of the impact of financing mechanisms on maternal health care in Australia
Source: BMC Public Health. 2019 Nov 21;19:1540. doi: 10.1186/s12889-019-7850-6 (PMC6873587; doi:10.1186/s12889-019-7850-6)
Supplement: Supplementary file 1 — Additional file 1. Documents included in the review. [file 12889_2019_7850_MOESM1_ESM.docx]

Additional file 1: Documents included in the review

| **Author/organisation** | **Date of manuscript** | **Type of manuscript** | | **Document title** | **Funding** | | **Pooling** | | | **Purchase** | | |  |
| --- | --- | --- | --- | --- | --- | --- | --- | --- | --- | --- | --- | --- | --- |
| Administrator National Health Funding Pool | 23-Feb-16 | Website: www.publichospitalfunding.gov.au | The role of the Administrator | | |  | |  | | |  | | |
| Administrator National Health Funding Pool | 1-Jun-17 | Annual report | 2016-17 Administrator National Health Funding Pool Annual Report | | |  | |  | | |  | | |
| Amanda Biggs, Parliament of Australia | 29-Oct-04 | Policy background brief | Medicare - Background Brief | | |  | |  | | |  | | |
| Australian Bureau of Statistics | 27-Mar-17 | Website: www.abs.gov.au | Private Health Insurance | | |  | |  | | |  | | |
| Australian Bureau of Statistics | 29-Oct-02 | Website: www.abs.gov.au | Private Medical Practitioners, Australia, 2002 | | |  | |  | | |  | | |
| Australian Government, Private Health Insurance Ombudsman | Mar-17 | Website: www.privatehealth.gov.au | Australian Government Private Health Insurance Rebate | | |  | |  | | |  | | |
| Australian Government, Private Health Insurance Ombudsman | 9-Jul-05 | Website: www.privatehealth.gov.au | Out of pocket expenses (gap cover) | | |  | |  | | |  | | |
| Australian Government | 2015 | Australian Government website | How Government works | | |  | |  | | |  | | |
| Australian Government | 26-Mar-14 | Australian Government Senate inquiry | Out-of-pocket costs in Australian healthcare | | |  | |  | | |  | | |
| Australian Government, Australian Prudential Regulation Authority | 6-Nov-17 | Australian Government Department Annual Financial Activities Report | The Operations of Private Health Insurers Annual Report 2016/17 | | |  | |  | | |  | | |
| Australian Government, Australian Prudential Regulation Authority | 16-Aug-17 | Australian Government Department Report | Private Health Insurance Medical Gap | | |  | |  | | |  | | |
| Australian Government, Australian Taxation Office | 29-Jun-17 | Website: www.ato.gov.au | Medicare levy reduction for low-income earners | | |  | |  | | |  | | |
| Australian Government, Australian Taxation Office | 29-Jun-17 | Website: www.ato.gov.au | Medicare levy surcharge | | |  | |  | | |  | | |
| Australian Government, Australian Taxation Office | 29-Jun-17 | Website: www.ato.gov.au | Medicare levy | | |  | |  | | |  | | |
| Australian Government, Department of Health | Nov-17 | Website: www.health.gov.au | Lifetime Health Cover | | |  | |  | | |  | | |
| Australian Government, Department of Health | 26-Oct-17 | Website: www.health.gov.au | Changes to MBS items for Obstetric Services Frequently Asked Questions | | |  | |  | | |  | | |
| Australian Government, Department of Health | 1-Jul-17 | Medicare Schedule | Medicare Benefits Schedule Book | | |  | |  | | |  | | |
| Australian Government, Department of Health | 2011 | Website: www.health.gov.au | Provision of maternity care | | |  | |  | | |  | | |
| Australian Government, Department of Health | 1-Dec-15 | Website: www.pbs.gov.au | Pharmaceutical Benefits Scheme (PBS) | | |  | |  | | |  | | |
| Australian Government, Department of Health | 1-Dec-15 | Website: www.pbs.gov.au | Patient Charges | | |  | |  | | |  | | |
| Australian Government, Department of Health | 11-Oct-17 | Website: www.pbs.gov.au | PBS Frequently asked questions | | |  | |  | | |  | | |
| Australian Government, Department of Health | 11-Nov-16 | Website: www.health.gov.au | Child and Maternal Health | | |  | |  | | |  | | |
| Australian Government, Department of Health | 2013 | www.health.gov.au | Eligible Midwives Questions and Answers | | |  | |  | | |  | | |
| Australian Government, Department of Health | 2009 | Senate inquiry | Submission to the Senate Standing Committee on Community Affairs for the Inquiry into the Health Insurance Amendment (Extended Medicare Safety Net) Bill 2009 | | |  | |  | | |  | | |
| Australian Government Department of Human Services | 2017 | Website: www.humanservices.gov.au | Medicare Bulk Billing | | |  | |  | | |  | | |
| Australian Institute of Health and Welfare | 13-Sep-16 | Australian Government Department National Annual Health Report | Australia's Health, 2016 | | |  | |  | | |  | | |
| Australian Institute of Health and Welfare | 7-May-08 | Australian Government Department National Annual Health Report | Australia's Health, 2008 | | |  | |  | | |  | | |
| Australian Institute of Health and Welfare | 2000 | National Maternal and Child Health Report | Australia’s mothers and babies | | |  | |  | | |  | | |
| Australian Institute of Health and Welfare | 2008 | National Maternal and Child Health Report | Australia’s mothers and babies | | |  | |  | | |  | | |
| Australian Institute of Health and Welfare | 2017 | National Maternal and Child Health Report | Australia’s mothers and babies 2016 – in brief | | |  | |  | | |  | | |
| Australian Institute of Health and Welfare | 2017 | National Annual Health Expenditure Report | Health expenditure Australia 2015-16 | | |  | |  | | |  | | |
| Australian Institute of Health and Welfare | 1-Jun-15 | Australian Government Department National Aboriginal and Torres Strait Islander Health Report | The health and welfare of Australia's Aboriginal and Torres Strait Islander peoples | | |  | |  | | |  | | |
| Australian Institute of Health and Welfare | July 2016 | Annual national hospital resources report | Hospital resources 2014-15: Australian hospital statistics | | |  | |  | | |  | | |
| Australian Institute of Health and Welfare | 17-Sept-14 | Literature review | Nomenclature for models of maternity care: literature reivew | | |  | |  | | |  | | |
| Australian Local Government Association | 2-Jul-05 | National Local Government report | Health and wellbeing | | |  | |  | | |  | | |
| Barry Burgan, the University of Adelaide | 2015 | Academic report | Funding a viable and effective health sector in Australia | | |  | |  | | |  | | |
| Bupa | 2017 | Private Health Insurance website | Bupa seeks to deliver gap free childbirth | | |  | |  | | |  | | |
| Consumers Health Forum of Australia | 7-Jan-16 | Position Statement | Preserving Consumer Choices Without Sacrificing the Principles of Universal Health Care | | |  | |  | | |  | | |
| Council of Australian Governments | 1-Jul-17 | Legislative document | National Health Reform Agreement | | |  | |  | | |  | | |
| Natasha Donnolley, Georgina Chambers, Kerryn Butler-Henderson, Michael Chapman and Elizabeth Sulivan | 2017 | Peer review publication in an academic journal | The Maternity Care Classification System – A validated system for classifying models of care | | |  | |  | | |  | | |
| Natasha Donnolley, Kerryn Butler-Henderson, Michael Chapman and Elizabeth Sullivan | 2016 | Peer review publication in an academic journal | The development of a classification system for maternity models of care | | |  | |  | | |  | | |
| Natasha Donnolley, Georgina Chambers, Kerryn Butler-Henderson, Michael Chapman and Elizabeth Sullivan | 2017 | Peer review publication in an academic journal | More than a name: Heterogeneity in characteristics of models of maternity care reported from the Australian Maternity Care Classification System validation study | | |  | |  | | |  | | |
| Natasha Donnolley, Georgina Chambers, Kerryn Butler-Henderson, Michael Chapman and Elizabeth Sullivan | 2017 | Peer review publication in an academic journal | A Validation study of the Australian Maternity Care Classification System | | |  | |  | | |  | | |
| Einarsdóttir, Kemp, A.b, Haggar, F.A.b, Moorin, R.E.b,c, Gunnell, A.S.d, Preen, D.B.b, Stanley, F.J.a, Holman, C.D.J.b | 23-Jul-12 | Peer review publication in an academic journal | Increase in caesarean deliveries after the Australian private health insurance incentive policy reforms | | |  | |  | | |  | | |
| Emily Callander & Haylee Fox | Dec-17 | Peer review publication in an academic journal | Changes in out-of-pocket charges associated with obstetric care provided under Medicare in Australia | | |  | |  | | |  | | |
| Independent Hospital Pricing Authority | 8-Aug-17 | Independent Government Agency Publication | Activity Based Funding | | |  | |  | | |  | | |
| J Hall | 6-Aug-15 | Peer review publication in an academic journal | Australian health care - The challenge of reform in a fragmented system | | |  | |  | | |  | | |
| Julie Smith | 1-Dec-01 | Peer review publication in an academic journal | Tax expenditures and Public Health Financing in Australia | | |  | |  | | |  | | |
| Madeline Taylor | Jul-12 | Peer review publication in an academic journal | Is it a levy, or is it a tax, or both? | | |  | |  | | |  | | |
| McLachlan HL, Forster DA, Davey MA, Farrell T, Gold L, Biro MA et al. | Nov-12 | Peer review publication in an academic journal | Effects of continuity of care by a primary midwife (caseload midwifery) on caesarean section rates in women of low obstetric risk: the COSMOS randomised controlled trial | | |  | |  | | |  | | |
| National Rural Health Alliance Ltd. | 31-Jul-17 | A Working Document to inform policy | The little book of rural health numbers | | |  | |  | | |  | | |
| Parliament of Australia | 29-Mar-17 | Parliamentary Communique | Value and affordability of private health insurance and out-of-pocket medical costs | | |  | |  | | |  | | |
| Australian Healthcare & Hospitals Association | 29-Mar-17 | Website: www.aph.gov.au | Value and affordability of private health insurance and out-of-pocket medical costs | | |  | |  | | |  | | |
| Richard Denniss | 1-Nov-05 | Peer review publication in an academic journal | Who Benefits from Private Health Insurance in Australia? | | |  | |  | | |  | | |
| Rosemary Bryant, Commonwealth of Australia | Feb-09 | Australian Government Report | Improving Maternity Services in Australia | | |  | |  | | |  | | |
| Sandall J, Soltani H, Gates S, Shennan A, Devane D | Feb-17 | Peer review publication in an academic journal | Midwife‐led continuity models versus other models of care for childbearing women | | |  | |  | | |  | | |
| Stephen Robson, Paula Laws and Elizabeth Sullivan | 4-May-09 | Peer review publication in an academic journal | Adverse outcomes of labour in public and private hospitals in Australia: a population-based descriptive study | | |  | |  | | |  | | |
| Shorten B, Shorten A | 2004 | Peer review publication in an academic journal | Impact of private health insurance incentives on obstetric outcomes in NSW hospitals | | |  | |  | | |  | | |
| SK Tracy, Welsh A, Hall B, Hartz D, Lainchbury A, Bisits A, et al. | 2014 | Peer review publication in an academic journal | Caseload midwifery compared to standard or private obstetric care for first time mothers in a public teaching hospital in Australia: a cross sectional study of cost and birth outcomes | | |  | | |  | | |  |  |
| SK Tracy, MB Tracy | Aug-03 | Peer review publication in an academic journal | Costing the cascade: estimating the cost of increased obstetric intervention in childbirth using population data | | |  | | |  | | |  |  |
| Stephen Duckett, Grattan Institute | Mar-13 | Think Tank Policy Report | Australia's bad drug deal | | |  | |  | | |  | | |
| David Richardson, the Australian Institute | May-17 | Think tank discussion paper | Time for a progressive Medicare levy | | |  | |  | | |  | | |
| The Royal Women's Hospital | 9-Jul-05 | Public Hospital Website | Pregnancy care & birthing options | | |  | |  | | |  | | |
| Kees Van Gool Elizabeth Savage Rosalie Viney Marion Haas Rob Anderson | 31-May-09 | Peer review publication in an academic journal | Who’s getting caught? An analysis of the Australian Medicare Safety Net | | |  | |  | | |  | | |
| Australian Institute of Health and Welfare | 13-Jun-19 | National annual report | Disease expenditure in Australia | | |  | |  | | |  | | |
|  |  |  |  | | |  | |  | | |  | | |
| Amanda Biggs | 2018 | Update on policy developments | Recent developments in federal government funding for public hospitals: a quick guide | | |  | |  | | |  | | |
| Australian Prudential Regulation Authority | 21-May-19 | Private health insurance statistics | Quarterly Private Health Insurance Statistics. March 2019 | | |  | |  | | |  | | |
| Stephen Duckett & Kristina Nemet | Jul-19 | Think Tank Working Paper | The history and purposes of private health insurance. | | |  | |  | | |  | | |

1. Donnolley N, Butler-Henderson K, Chapman M, Sullivan E. The development of a classification system for maternity models of care. Health Information Management Journal. 2016;45(2):64-70.

2. Australian Institute of Health and Welfare. Health expenditure Australia 2015-16. Canberra, Australia: AIHW; 2017.
